# Enhanced Sensing Mechanism Based on Shifting an Exceptional Point

**DOI:** 10.34133/research.0260

**Published:** 2023-10-31

**Authors:** Xuan Mao, Guo-Qing Qin, Hao Zhang, Bo-Yang Wang, Dan Long, Gui-Qin Li, Gui-Lu Long

**Affiliations:** ^1^ Department of Physics, State Key Laboratory of Low-Dimensional Quantum Physics, Tsinghua University, Beijing 100084, China.; ^2^ Beijing Institute of Radio Measurement, The Second Academy of China Aerospace Science and Industry Corporation (CASIC), Beijing 100854, China.; ^3^ Purple Mountain Laboratories, Nanjing 211111, China.; ^4^ Frontier Science Center for Quantum Information, Beijing 100084, China.; ^5^ Beijing Academy of Quantum Information Sciences, Beijing 100193, China.

## Abstract

Non-Hermitian systems associated with exceptional points (EPs) are expected to demonstrate a giant response enhancement for various sensors. The widely investigated enhancement mechanism based on diverging from an EP should destroy the EP and further limits its applications for multiple sensing scenarios in a time sequence. To break the above limit, here, we proposed a new enhanced sensing mechanism based on shifting an EP. Different from the mechanism of diverging from an EP, our scheme is an EP nondemolition and the giant enhancement of response is acquired by a slight shift of the EP along the parameter axis induced by perturbation. The new sensing mechanism can promise the most effective response enhancement for all sensors in the case of multiple sensing in a time sequence. To verify our sensing mechanism, we construct a mass sensor and a gyroscope with concrete physical implementations. Our work will deepen the understanding of EP-based sensing and inspire designing various high-sensitivity sensors in different physical systems.

## Introduction

Non-Hermitian quantum systems present attractive properties such as exceptional points (EPs) [1–6] at which 2 or more eigenvalues and the corresponding eigenstates coalesce simultaneously [7]. The existence of EPs has been verified in various physical systems such as optomechanical systems [8–10], coupled atom–cavity systems [11], and plasmonic systems [12–14]. The exotic properties of non-Hermitian systems [15–18] open potential possibilities for diverse advanced applications including phonon lasers [9,19–21], chiral mode conversion [8,22–26], and enhanced sensing [3,4,27–32]. The response enhancement at EPs has been widely investigated in microcavities [3,4,29,33], optomechanical systems [31,34,35], and circuits [28,36–38].

Owing to the complex response near an *n*th-order EP, any small perturbation destroys the degeneracy that results in an *n*th-root response for the frequency splitting [4]. For sufficiently small perturbation, the splitting is much larger than observed in conventional schemes where the response is proportional to the perturbation strength. The giant enhancement of EPs has been verified both theoretically and experimentally in various detection schemes [3,4,32,39–41] ranging from mass sensor [31] to nanoparticle detector [4,29,32], from magnetometer [34,39] to gyroscope [3,33,35,42–44]. Not only the presence of perturbation will cause the frequency splitting, but also the degeneracy of the linewidths disappears after introducing perturbation. The EP condition is totally destroyed by the perturbation, and the EP no longer exists which will limit the enhancement for multiple sensing schemes in a time sequence. In this case, it is necessary to develop an EP condition nondemolition enhanced sensing mechanism to break the limit.

In this paper, we propose a novel EP-based enhanced sensing mechanism that relies on the shift of an EP in contrast to the conventional method of diverging from an EP. For the enhanced mechanism based on diverging from an EP, the perturbation breaks the EP condition, and it further deteriorates the EP enhance performance for implementing multiple sensing in a time sequence. Compared with diverging from an EP mechanism, the enhanced sensing mechanism based on shifting an EP demonstrates a slight shift along the parameter axis induced by the perturbation resulting in a remarkable enhancement for the frequency splitting. For the shift of an EP, there exists an EP in parameter space after perturbation, and one can prepare the system back to an EP state that promises the most effective enhancement for every sensing. Furthermore, we propose a mass sensor and a gyroscope scheme based on shifting an EP to verify the enhanced sensing mechanism. This work opens up paths to design ultrasensitive sensors and verifies potential applications in various fields including precision measurement and quantum metrology.

## Results

### Two enhanced sensing mechanisms based on an EP

**Fig. 1.**
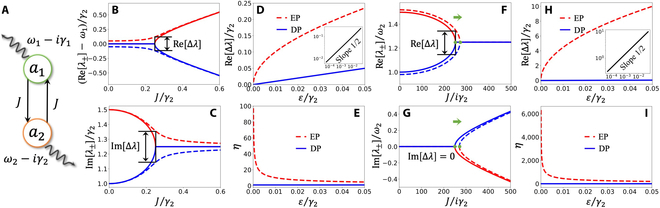
The general model. (A) The schematic of the coupled 2-level system. The real parts (B) and the imaginary parts (C) of the eigenvalues versus *J* before (solid lines) and after (dashed lines) introducing the perturbation for the enhanced sensing mechanism based on diverging from an EP. *ω*_1_/*γ*_2_ = *ω*_2_/*γ*_2_ = 1, 500, *γ*_1_/*γ*_2_ = 1.5, and *ε*/*γ*_2_ = 0.05. (D) Re[Δ*λ*] versus the strength of the perturbation *ε* in the case of diverging from an EP (red dashed line) and a DP (blue solid line). The inset indicates [ReΔ*λ*] versus *ε* in the case of diverging from an EP in a logarithmic scale. (E) The enhancement factor of sensors based on diverging from an EP (red dashed line) and a DP (blue solid line). *ω*_1_/*γ*_2_ = *ω*_2_/*γ*_2_ = 1, 500, *γ*_1_/*γ*_2_ = 1.5, and *J*/*γ*_2_ = 0.25. The real parts (F) and the imaginary parts (G) of the eigenvalues versus *J* before (solid lines) and after (dashed lines) introducing the perturbation for the enhanced sensing mechanism based on shifting an EP. The green dots represent the location of EPs. *ω*_1_/*γ*_2_ = 1, 500, *ω*_2_/*γ*_2_ = 1, 000, *γ*_1_/*γ*_2_ = 1, and *ε*/*γ*_2_ = 20. (H) Re[Δ*λ*] versus the strength of the perturbation *ε* in the case of shifting an EP (red dashed line) and a DP (blue solid line). The inset indicates Re[Δ*λ*] versus *ε* in the case of shifting an EP in a logarithmic scale. (I) The enhancement factor of sensors based on shifting an EP (red dashed line) and a DP (blue solid line). *ω*_1_/*γ*_2_ = 1, 500, *ω*_2_/*γ*_2_ = 1, 000, *γ*_1_/*γ*_2_ = 1, and *J*/*iγ*_2_ = 250.

Considering a 2-level coupled system composed of 2 modes represented by *a*_1_ and *a*_2_ as illustrated by the inset of Fig. 1A, the dynamical equations of the general coupled system can be written as

$$\frac{d}{\mathrm{dt}}\left(\begin{array}{c} a_{1}\\ a_{2} \end{array}\right)=-i\left(\begin{array}{cc} \omega_{1}-i\gamma_{1} & J\\ J & \omega_{2}-i\gamma_{2} \end{array}\right)\left(\begin{array}{l} a_{1}\\ a_{2} \end{array}\right),$$ (1)

where *t* can be the evolution time and the propagation distance. The resonance frequency and the decay of *a*_1_ (*a*_2_) are represented by *ω*_1_ (*ω*_2_) and *γ*_1_ (*γ*_2_), respectively. *J* denotes the coupling coefficient, and it can be real or complex. For the purpose of conciseness, we only consider the reciprocal coupling case and the similar conclusion can be obtained for the nonreciprocal coupling. Assuming that the solutions have the harmonic form *a*_1, 2_ = *a*_1, 2_*e*^−*iλt*^, the eigenvalues of the coupled system can be expressed as

$$\lambda_{\pm }=\omega_{\text{mean}}-i\gamma_{\text{mean}}\pm \sqrt{{\left(\omega_{\text{diff}}+i\gamma_{\text{diff}}\right)}^{2}+J^{2}}.$$ (2)

*ω*_mean_ = (*ω*_1_ + *ω*_2_)/2 and *γ*_mean_ = (*γ*_1_ + *γ*_2_)/2 represent the mean values of the resonance frequency and the loss. *ω*_diff_ = (*ω*_1_ − *ω*_2_)/2 and *γ*_diff_ = (*γ*_1_ − *γ*_2_)/2 are the differences of the frequency and the decay. It can be indicated that when the parameter values meet the requirement ±*iJ* = *ω*_diff_ + *iγ*_diff_, the system is prepared on an EP state.

The perturbation introduced to the sensor systems can induce frequency shift [3,31], linewidth broadening [45,46], or both. For the sake of simplicity, we consider the case that the perturbation only induces frequency shift to illustrate the difference between the 2 enhanced sensing mechanisms based on an EP.

If the coupling coefficient *J* is a real number and the enhanced sensing mechanism is well known as the eigenvalues diverging from an EP, the enhanced sensing mechanism based on the eigenvalues diverging from an EP after the perturbation *ε* is illustrated by Fig. 1B and C. The solid lines and the dashed lines represent the evolution of the 2 eigenvalues along with the system parameter *J* before and after introducing the perturbation. Usually, before introducing perturbation, the sensing system is prepared near an EP and one can monitor the frequency shift Re[Δ*λ*] and the linewidth change Im[Δ*λ*] after the perturbation. According to the frequency shift, the strength of the perturbation *ε* can be indicated. Due to the singularity of EPs, the frequency shifts of the eigenvalues obtain *ε*^1/2^ (the red dashed line in Fig. 1D) enhancement comparing to the diabolic point (DP) (the blue solid line in Fig. 1D) in the condition of *ε* ≪ 1. In contrast to EP, some microcavities support modes also with degenerate eigenvalues, but the associated eigenvectors can always be chosen to be orthogonal to each other, also known as DP. For the traditional cavity-based sensing schemes, the DP is utilized and the perturbation-induced frequency shift response is proportional to the perturbation strength, which is shown by the blue solid line in Fig. 1D. The inset of Fig. 1D indicates the frequency shift value is proportional to *ε*^1/2^ as the slope is 1/2 in a logarithmic scale. To demonstrate the enhancement of EPs, the enhancement factor can be defined as

η=∣ReΔλε∣. (3)

Figure 1E illustrates the enhancement factor of an EP (the red dashed line) and a DP (the blue solid line) versus the perturbation strength. It can be indicated that an EP enhance performance is much better in the weak perturbation regime, which is consistent with the analysis above. It can be inferred that the EP condition is destroyed by the perturbation and the EP no longer exists in the parameter space for the enhanced sensing mechanism based on diverging from an EP. In the case of implementing multiple sensing in a time sequence, the enhancing performance deteriorates as the number of sensingincreases as every next detection is conducted on the basis of being destroyed by the previous detection.

The shift of an EP mechanism requires the coupling coefficient is an imaginary number and can also exhibit ε^1/2^ enhancement. The imaginary coupling between the 2 modes have been experimentally realized in dissipative coupled systems including atomic systems [47,48], optical systems [3,49,50], mechanical systems [9], circuits [51], and thermal materials [52]. Under some specific parameter values, the existence of the EP can be maintained along with a shift in the parameter space after the perturbation which is denoted by Fig. 1F and G. Due to the topological structure near an EP, the shift of an EP will lead to similar enhancement results to the sensing performance based on the eigenvalues diverge. The response of eigenvalues to the strength of perturbation and the enhancement factor of shifting an EP mechanism are demonstrated in Fig. 1H and I, respectively. On the other hand, the linewidth changes of the 2 eigenstates demonstrate different behaviors. In the case of an EP shift sensing, the 2 linewidths of the eigenstates stay the same as before the perturbation, which may further improve the enhancement performance in the perspective of experiments. The unbroadened linewidths after the perturbation may improve the precision of dispersion measurement. Another interesting point is there also exists an EP in the parameter space after the perturbation for the sensors based on shifting an EP which is an EP condition nondemolition enhanced sensing mechanism. One can prepare the system back to an EP state by tuning corresponding parameters after introducing the perturbation. In the case of the influence of the perturbation to the sensor that is hard to eliminate such as mass sensor and nanoparticle sensing, it is a challenging task to separate the deposited mass or nanoparticle and the sensing system, and the sensors based on shifting an EP will always obtain the most efficient enhancement as the system can be prepared back to an EP before the next sensing. Thus, for the task of implementing multiple sensing in a time sequence, sensors based on shifting an EP will always demonstrate the most effective EP enhancement for every detection.

As a conclusion, we demonstrate that the 2 enhanced sensing mechanisms based on an EP for the perturbation only induces frequency shift, and the case that the linewidth broadening induced by the perturbation is similar to the case we consider here. The conclusions for the 2 different systems can be found in Table.

**Table.** The enhanced sensing mechanisms for different perturbations and coupling systems.

Perturbation induces	Real coupling systems	Imaginary coupling systems
Frequency shift	Diverging from an EP	Shift of an EP
Linewidth broadening	Shift of an EP	Diverging from an EP

### Mass sensor based on shifting an EP

**Fig. 2.**
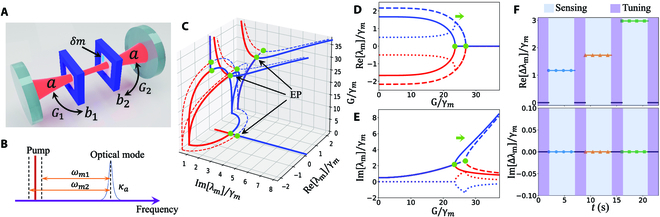
Mass sensor based on shifting an EP. (A) The mass sensor schematic. (B) The pump scheme in frequency domain. (C) The evolution of the eigenvalues under different optomechanical coupling strength *G*. EPs are denoted by the green dots. (D and E) The real parts and the imaginary parts of the mass sensor system versus *G*. The solid lines and the dashed lines represent before and after introducing a deposited mass, respectively. The dotted lines are the differences between the eigenvalues before and after the perturbation. *δω* = 0.0005*ω*_1_. (F) The differences of the real parts and the imaginary parts for implementing multiple mass sensing in a time sequence. The corresponding frequency shifts are *δω* = 0.0001*ω*_1_, 0.0002*ω*_1_, and 0.0005*ω*_1_, respectively. The other parameters are *ω*_*m*1_/2*π* = 6 GHz, *ω*_*m*2_/2*π* = 6.01 GHz, *γ*_*m*1_/2*π* = *γ*_*m*2_/2*π* = *γ_m_*/2*π* = 3 MHz, and *κ_a_*/2*π* = 2 GHz.

Optomechanics [53] has been a promising platform to investigate fundamental phenomena [54–58] and applied science [59–64]. Benefiting from abundant manipulation methods, realization of ultrasensitive sensors operating at EPs in optomechanical systems has gained wide attention over the last few decades. To illustrate the enhanced mechanism based on shifting an EP, here, we propose an optomechanical mass sensor. The mass sensor system as illustrated by Fig. 2A consists of 2 mechanical vibrators *b*_1_ and *b*_2_ coupling to the same optical field *a* with frequency *ω_a_* and loss *κ_a_*. The Hamiltonian of the system is (*ℏ* = 1)

$$\begin{array}{c} H_{m}=\Sigma_{j=1,2}‍[\omega_{\mathrm{mj}}b_{j}^{†}b_{j}+g_{j}a^{†}a\left(b_{j}+b_{j}^{†}\right)]+\\ \omega_{a}a^{†}a+i\sqrt{\kappa_{\mathrm{ex}}}\epsilon \left(a^{†}e^{-i\omega_{d}t}-ae^{i\omega_{d}t}\right) \end{array}$$ (4)

The mechanical resonator *b_j_* is characterized by the frequency *ω_mj_* and the damping rate *γ_mj_* (*j* = 1, 2). *g_j_* is the single-photon optomechanical coupling strength between the mechanical mode *b_j_* and the optical mode *a*. *κ*_ex_ represents the coupling loss. Figure 2B represents the driving scheme of the mass sensor in frequency domain. The pump laser owns the driving frequency *ω_d_* and the amplitude *ϵ*. Following the standard optomechanical linearization procedure and adiabatically eliminate the optical field, one can obtain the effective Hamiltonian of the system in the matrix form

$$H_{m}^{\text{eff}}=\left(\begin{array}{ll} \Omega_{0}-i\left(\frac{\gamma_{m1}}{2}+\frac{2G_{1}^{2}}{\kappa_{a}}\right) & -i\frac{2G_{1}G_{2}}{\kappa_{a}}\\ -i\frac{2G_{1}G_{2}}{\kappa_{a}} & -\Omega_{0}-i\left(\frac{\gamma_{m2}}{2}+\frac{2G_{2}^{2}}{\kappa_{a}}\right) \end{array}\right),$$ (5)

where Ω_0_ = (*ω*_*m*1_ − *ω*_*m*2_)/2. $G_{i}=g_{i}\sqrt{n}$ is the effective optomechanical coupling strength with *n* denoting the intracavity photon number. *κ_a_* = *κ_ex_* + *κ*_0_ represents the total loss rate of the optical mode. *κ*_0_ indicates the intrinsic loss of the cavity mode. For convenience, we can set *G*_1_ = *G*_1_ = *G* and *γ*_*m*1_ = *γ*_*m*2_ = *γ_m_*. The eigenvalues of $H_{m}^{\mathrm{eff}}$ are given by

$$\lambda_{m\pm }=-i\left(\gamma_{m}/2+\Gamma \right)\pm \sqrt{\Omega_{0}^{2}-\Gamma^{2}},$$ (6)

where Γ = 2*G*^2^/*κ_a_*. Note that the Hamiltonian form in Eq. 5 satisfies anti-parity time (PT) symmetry with an EP at Γ =  ∣ Ω_0_∣. The evolution of the eigenvalues under different optomechanical coupling strengths are illustrated by the solid lines in Fig. 2C to E. Note that the locations of EPs are marked by the green dots in Fig. 2C.

For a traditional mass sensor, the relationship between the deposited mass *δm* and the caused frequency shift *δω* can be given by [65]

$$\delta \omega =\frac{\omega_{m2}}{2m}\delta m=R\delta m.$$ (7)

*R* = *ω*_*m*2_/2*m* represents the mass responsivity. *ω*_2_ and *m* are the resonant frequency and the effective mass of the mechanical resonator supporting the deposition.

After depositing mass *δm* into the mechanical resonator *b*_2_, the effective Hamiltonian of the system is

$$H_{m}^{{\mathrm{eff}}^{\prime }}=\left(\begin{array}{ll} \Omega_{0}^{\prime }-i\left(\frac{\gamma_{m1}}{2}+\frac{2G_{1}^{2}}{\kappa_{a}}\right) & -i\frac{2G_{1}G_{2}}{\kappa_{a}}\\ -i\frac{2G_{1}G_{2}}{\kappa_{a}} & -\Omega_{0}^{\prime }-i\left(\frac{\gamma_{m2}}{2}+\frac{2G_{2}^{2}}{\kappa_{a}}\right) \end{array}\right),$$ (8)

where $\Omega_{0}^{\prime }=\left(\omega_{m1}-\omega_{m2}-\delta \omega \right)/2$ . After introducing the mass, the EP will move from the former point in the parameter space as Fig. 2C to E shown. The dashed lines demonstrate the evolution of the eigenvalues after the perturbation. In Fig. 2D and E, the dotted lines show the difference between the eigenvalues before and after, i.e., Re[*λ_m_*]_after_ −  Re [*λ_m_*]_before_ and Im[*λ_m_*]_after_ −  Im [*λ_m_*]_before_. It can be noticed that the deposited mass causes the shift of the EP and further lead to the largest frequency shift near the EP. Meanwhile, the introduction of the deposited mass will not change the bandwidth of the eigenstate, which theoretically boosts the sensitivity enhancement for EP-based sensors.

One can implement multiple mass sensing in a time sequence utilizing the mass sensor based on shifting an EP as it can be tuned back to an EP after each sensing period. Figure 2F demonstrates the differences of the real parts and the imaginary parts. There are 2 periods, sensing period and tuning period, during the time sequence mass sensing. During the sensing periods, the frequency shifts obtain the most effective enhancement and present mass-dependent response. The corresponding frequency shifts due to the deposited mass are *δω* = 0.0001*ω*_1_, 0.0002*ω*_1_, and 0.0005*ω*_1_, respectively. During the tuning periods, the system can be prepared back to an EP by tuning the optomechanical coupling strength. Note that no matter the system experiences sensing periods or tuning periods, the linewidths of the sensor are always the same as the unperturbed system as shown in Fig. 2F.

### Gyroscope based on shifting an EP

**Fig. 3.**
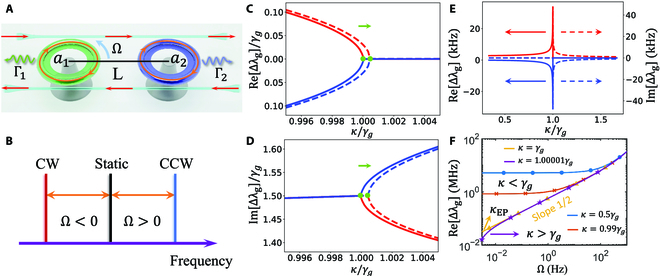
Gyroscope based on shifting an EP. (A) The gyroscope schematic. (B) The Sagnac frequency shift due to the introduction of rotation. The black line represents the static resonant frequency of the optical mode *a*_1_. The red (blue) frequency shift is corresponding to the CW (CCW) rotation direction, which is denoted by the red (blue) line. (C) The real parts and (D) the imaginary parts of the eigenvalues of the system as a function of *κ*/*γ_g_*. The solid (dashed) lines are corresponding to the case without (with) a rotation. Ω = 0.1 Hz. (E) The differences of the real parts and the imaginary parts of the eigenvalues before and after rotation under different values of *κ*. Ω = 0.1 Hz. (F) Dependence of frequency splitting on the rotation frequency Ω in the case of anti-PT broken phase (*κ* < *γ_g_*), anti-PT symmetry phase (*κ* > *γ_g_*), and at an EP (*κ*_EP_). The other parameters are *n* = 1.44, *R* = 9 mm, *c* = 3 × 10^8^ m/s, *λ* = 1, 550 nm, and Ω_−_ = Γ_1_ = Γ_2_ = *γ_g_* = 3 MHz.

The gyroscope scheme based on shifting an EP is illustrated by Fig. 3A. Two whispering-gallery-mode (WGM) cavities are coupled to 2 drop-filter waveguides with center-to-center distance *L*. On the basis of the 2 resonator modes Ψ = (*a*_1_, *a*_2_)^⊤^, the effective Hamiltonian in matrix form can be expressed as [66]

$$H_{g}^{\mathrm{eff}}=\left(\begin{array}{ll} \omega_{a1}-i\frac{\gamma_{\mathrm{eff}1}}{2} & -ie^{\mathrm{i\varphi}}\sqrt{\kappa_{1}\kappa_{2}}\\ -ie^{\mathrm{i\varphi}}\sqrt{\kappa_{1}\kappa_{2}} & \omega_{a2}-i\frac{\gamma_{\mathrm{eff}2}}{2} \end{array}\right),$$ (9)

where *ω*_*a*1_ and *ω*_*a*2_ are the resonance frequencies of the 2 WGM resonators. *γ*_effj_ = Γ*_j_* + 2*κ_j_* denotes the effective loss and Γ*_j_* represents the intrinsic loss rate in the *j*th resonator. *κ_j_* is the coupling rate between the *j*th resonator and the drop-filter waveguide. The phase delay factor is *φ* = 2*πn_w_L*/*λ*, with *n_w_* being the refractive index of waveguides and *λ* representing the wavelength of the light.

Benefiting from the tunability of the phase delay, the indirectly coupling system can exhibit anti-PT symmetry [9,47,52,67]. Without losing of generality, we can assume the 2 resonators have the equal losses Γ_1_ = Γ_2_ and the waveguide-resonator coupling rates are equal *κ*_1_ = *κ*_2_ = *κ*. Thereby, the effective Hamiltonian in anti-PT symmetry form is

$$H_{g}^{{\mathrm{eff}}^{\prime }}=\left(\begin{array}{cc} \Omega_{-}-i\left(\frac{\Gamma_{1}+\Gamma_{2}}{4}+\kappa \right) & \mathrm{i\kappa}\\ \mathrm{i\kappa} & -\Omega_{-}-i\left(\frac{\Gamma_{1}+\Gamma_{2}}{4}+\kappa \right) \end{array}\right).$$ (10)

Here, Ω_−_ = (*ω*_*a*1_ − *ω*_*a*2_)/2. When introducing rotation frequency into the first WGM resonator, the resonance frequency experiences a Sagnac frequency shift associated with the rotation direction

$$\Delta_{\mathrm{Sag}}=\frac{\mathrm{nR}\Omega \omega_{a1}}{c}\left(1-\frac{1}{n^{2}}-\frac{\lambda }{n}\frac{dn}{d\lambda }\right),$$ (11)

where *n* and *R* are the refractive index and the radius of the resonator, respectively. *c* denotes the speed of the light, and the dispersion term d*n*/d*λ* is relatively small in typical materials (∼1%) [68]. For convenience, we define the sign of the rotation frequency depending on the rotation direction. As shown in Fig. 3A and B, in the case of counterclockwise (CCW) rotation the sign of the rotation frequency is defined as positive associating with a positive Sagnac frequency shift and vice versa. For the case gyroscope experience clockwise (CW) rotation, the resonant frequency of *a*_1_ has a red shift as indicated in Fig. 3B. Due to the definition of the sign of the rotation frequency, one can simply add the perturbation matrix into Eq. 10 after introducing rotation

$$\begin{array}{c} H_{g}^{{\mathrm{eff}}^{\prime \prime }}=\left(\begin{array}{cc} \Omega \_+\Delta_{\text{Sag}}-i\left(\frac{\Gamma_{1}+\Gamma_{2}}{4}+\kappa \right) & i\kappa \\ i\kappa & -\Omega \_-i\left(\frac{\Gamma_{1}+\Gamma_{2}}{4}+\kappa \right) \end{array}\right) \end{array}$$ (12)

And the eigenvalues of the effective Hamiltonian are

$$\lambda_{g\pm }=\frac{\Delta_{\mathrm{Sag}}}{2}-i\left(\frac{\Gamma_{1}+\Gamma_{2}}{4}+\kappa \right)\pm \sqrt{{\left(\frac{\Delta_{\mathrm{Sag}}+2\Omega_{-}}{2}\right)}^{2}-\kappa^{2}}.$$ (13)

It is clear that no matter the system experiences rotation or not, there is always a threshold coupling strength at (Δ_Sag_ + 2Ω_−_)/2. The real parts and the imaginary parts of *λ*_*g*±_ are illustrated by Fig. 3C and D. In the absence of the rotation, there are 3 regimes for different values of *κ* and the threshold is *κ*_EP_ = Ω_−_ = *γ_g_*. In the domain of *κ* < *γ_g_*, the eigenstates share the same linewidth while possessing different resonance frequency and the system is in the anti-PT broken phase. In the case of *κ* > *γ_g_*, the 2 eigenvalues have equal real part and different imaginary parts, which indicates that the system is in the anti-PT symmetry phase. The EP is the phase transition point of anti-PT symmetry phase and anti-PT broken phase. It is indicated that the EP is shifting along the parameter axis *κ*/*γ_g_* after introducing a rotation. With the presence of the rotation, one can tune the system back to an EP state by adjusting the distance between the waveguides and the WGM cavities. Figure 3E shows the differences of the real parts and the imaginary parts of the eigenvalues before and after rotation, i.e., Re[*λ_g_*]_after_ −  Re [*λ_g_*]_before_ and Im[*λ_g_*]_after_ −  Im [*λ_g_*]_before_. The solid lines and the dashed lines denote the real parts and the imaginary parts, respectively. One can see near an EP that the system demonstrates a great frequency splitting and meanwhile maintains the linewidth.

After introducing rotation into the system, the reactions of the frequency splitting Re[Δ*λ*] =  ∣  Re[*λ*_*g*+_] −  Re[*λ*_*g*−_]∣ in 3 regimes are not the same. Figure 3F illustrates the logarithmic behavior of the frequency splitting under different rotations in the case of anti-PT symmetry phase (*κ* > *γ_g_*), anti-PT broken phase (*κ* < *γ_g_*), and at the EP (*κ*_EP_). In the case of the anti-PT broken phase, the frequency splitting is largest while the slope is smallest for the same perturbation strengths. However, for a small rotation frequency, the slope of the response at an EP is 1/2, which is predicted by the perturbation theory [4]. For a relatively large rotation frequency, the perturbation theory no longer works, and therefore, the slope of the response is larger than 1/2.

## Conclusion

We propose a novel enhanced sensing mechanism based on perturbation-induced shift of an EP in contrast to the widely investigated method of diverging from an EP. Diverging from an EP mechanism destroys the EP condition in the parameter space and obtains *ε*^1/2^ enhancement in the weak perturbation regime. In this case, the EP condition is totally destroyed by the perturbation and the EP no longer exists, which will limit the enhancement for multiple sensing schemes in a time sequence. To overcome the challenge, we propose the EP condition nondemolition enhanced sensing mechanism based on shifting an EP, which demonstrates a slight shift along the parameter axis induced by the perturbation and also exhibits remarkable enhancement. The linewidths will maintain after the perturbation for the EP shift mechanism, which may improve the precision of dispersion measurement. To implement the EP shift sensing mechanism, we construct a mass sensor and a gyroscope sensing scheme for the mechanical modes and the optical mode pair, respectively. Combining other enhancing methods such as exceptional surfaces [30,69,70] and nonlinearity [38,71,72], the performance of sensors will be further improved. The enhanced sensing mechanism proposed in this paper provides a comprehensive understanding of EPs enhancing and may inspire technological development in various schemes.

## Methods

In our numerical calculation, the frequencies and the linewidths of the modes are corresponding to the real parts and the imaginary parts of the eigenvalues of the system. These are crucial to the demonstration of EP and the influence of perturbation. For different types of perturbation, one can calculate the frequency and the linewidth of the system before and after introducing perturbation. The perturbation strength can be inferred by monitoring the corresponding changes.

## Data Availability

All data relevant to this work are available in the manuscript and supporting information. The data are available from the corresponding author upon reasonable request.
